# Community-Based Screening for Hepatitis B and C Infectivity Using Two Quantitative Antigens to Identify Endemic Townships

**DOI:** 10.3390/v14020304

**Published:** 2022-02-01

**Authors:** Wei-Cheng Huang, Yu-Chen Lin, Po-Ju Chen, Nien-Tzu Hsu, Chia-Ling Tu, Te-Sheng Chang, Chao-Hung Hung, Kwong-Ming Kee, Wen-Hua Chao, Sheng-Nan Lu

**Affiliations:** 1 Department of Geriatric, Chang Gung Memorial Hospital Chiayi Branch, Puzi 61363, Taiwan; rolando@cgmh.org.tw; 2 Department of Family Medicine, Kaohsiung Chang Gung Memorial Hospital, Kaohsiung 833253, Taiwan; thesmartesteverseen@gmail.com; 3 Chiayi County Health Bureau, Taibao 60044, Taiwan; cyhd235@cyshb.gov.tw (Y.-C.L.); chao624@cyshb.gov.tw (W.-H.C.); 4 Biostatistics Center, Kaohsiung Chang Gung Memorial Hospital, Kaohsiung 833253, Taiwan; e19911221@gmail.com; 5 Department of Gastroenterology and Hepatology, Chang Gung Memorial Hospital Chiayi Branch, Puzi 61363, Taiwan; gicltu@gmail.com (C.-L.T.); cgmh3621@cgmh.org.tw (T.-S.C.); 6 College of Medicine, Chang Gung University, Taoyuan 333323, Taiwan; chh4366@yahoo.com.tw (C.-H.H.); kee.kkm@gmail.com (K.-M.K.); 7 Division of Hepato-Gastroenterology, Department of Internal Medicine, Kaohsiung Chang Gung Memorial Hospital, Kaohsiung 833253, Taiwan; 8 Taiwan National Hepatitis C Program Office, Ministry of Health and Welfare, Taipei 115204, Taiwan

**Keywords:** elimination, endemic, hepatitis C virus, quantitative HBsAg, hepatitis C core antigen

## Abstract

Screening and linkage to care are essential to achieve viral hepatitis elimination before 2030. The accurate identification of endemic areas is important for controlling diseases with geographic aggregation. Viral activity drives prognosis of chronic hepatitis B and hepatitis C virus infection. This screening was conducted in Chiayi County from 2018–2019. All residents aged 30 years or older were invited to participate in quantitative HBsAg (qHBsAg) and HCV Ag screening. Among the 4010 participants (male:female = 1630:2380), the prevalence of qHBsAg and HCV Ag was 9.9% (396/4010) and 4.1% (163/4010), respectively. High-prevalence townships were identified, three for qHBsAg > 15% and two for HCV Ag > 10%. The age-specific prevalence of qHBsAg was distributed in an inverse U-shape with a peak (16.0%, 68/424) for subjects in their 40 s; for HCV, prevalence increased with age. Concentrations of qHBsAg < 200 IU/mL were found in 54% (214/396) of carriers. The rate of oral antiviral treatment for HCV was 75.5% (114/151), with subjects younger than 75 years tending to undergo treatment (85.6% vs. 57.4%, *p* < 0.001). QHBsAg and HCV Ag core antigens can reflect the concentration of the viral load, which serves as a feasible screening tool. Using quantitative antigen screening for hepatitis B and C in community-based screening, two hyperendemic townships were identified from an endemic county.

## 1. Introduction

Chronic viral hepatitis infection, including Chronic hepatitis B virus (HBV) and hepatitis C virus (HCV) infections, is a major disease worldwide. The World Health Organization (WHO) aims to eliminate viral hepatitis before 2030 [1]. In Taiwan, HBV infection is endemic in a non-vaccinated cohort. Although successful universal vaccination has been introduced, chronic HBV infection remains a health problem in the non-vaccinated cohort of those born before 1984 [2].

The prevalence of anti-HCV antibodies has been reported to be approximately 4–5%. A unique epidemiological characteristic of HCV infection is the geographic aggregation of people born before 1960 [3]. For the allocation priority of HCV control, accurate epidemiological information is necessary to determine areas with a high prevalence. Since the national screening project for HBV and HCV was launched in late 2020, the map has used seven nationwide hepatitis-related surrogates rather than the prevalence of anti-HCV. Although a precise hepatitis C prevalence map is urgently required, a nationwide township-specific map of liver disease risk was published in 2018 [4].

In most screening programs, HBV is screened using qualitative HBsAg and anti-HCV; however, neither marker indicates viral activity. Viral activity drives the outcome of chronic HBV [5] and HCV [6] infections and is also a determinant of antiviral treatment [7,8]. Although HBV DNA and HCV RNA are the gold standards for viral activity, quantitative antigen tests partially representing viral activity [9,10] may be more feasible for mass screening. In our community-based study, quantitative HBsAg (qHBsAg) levels of 23% of carriers were <8 IU/mL, and all HBV DNA levels were less than 2000 IU/mL [11]. This information is useful, especially in the elderly population. HBsAg clearance is age-dependent and related to long-term prognosis [12]. The anti-HCV test indicates those who are infected rather than infectious, and approximately 60–80% of the anti-HCV-positive subjects were positive for HCV RNA in a treatment-naïve population based on their natural history. Although the percentage of viremia should be lower in treatment-available populations, antiviral therapy, including interferon-based therapy, was launched an extensive time ago and has been reimbursed since 2003 [13]. Screening for viremia should be mandatory in the era of direct-acting antiviral agents (DAAs). The HCV core antigen (HCV Ag) may be a surrogate for HCV RNA [10,14]. Our community-based study showed that both positive and negative predictive values were >98% [15]. The cost-benefit of a single HCV Ag test appears to be better than that of the anti-HCV reflex HCV RNA test in community-based screening [14]. The aim of this study was to establish the baseline prevalence of HBV and HCV activity, identify the endemic areas, illustrate the experience of two quantitative antigens, and discuss their feasibility in hepatitis B and C screening.

## 2. Materials and Methods

**Figure 1:**
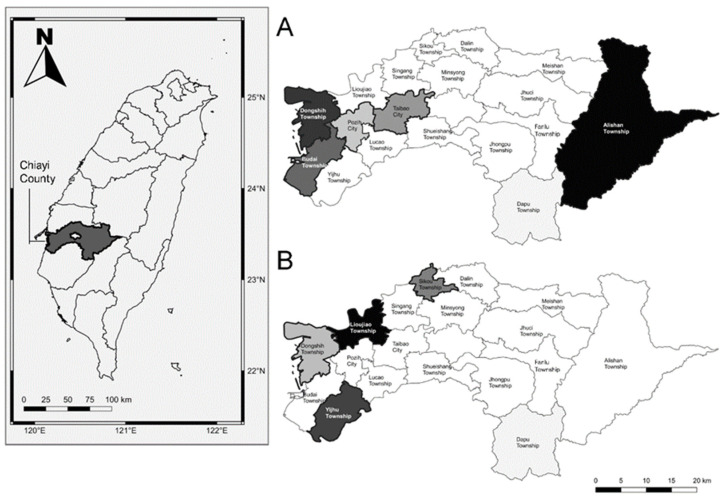
Location of the study county and endemic townships of (**A**) hepatitis B and (**B**) hepatitis C.

Chiayi County is located in the southwestern region of Taiwan and includes 18 townships (Figure 1). The incidence and mortality of primary liver cancer always rank in the top three among all 22 cities/counties [16]. Chiayi has been reported to have an occurrence of endemic chronic HCV infection [3,17], and although local government and non-governmental organizations have conducted small-scaled community screenings, there are no county-wide prevalence data available.

From October 2018 to May 2019, this community-based screening was conducted in Chiayi County, involving 17 out of 18 townships, excluding Dapu Township (Figure 1). The primary health care center of each township performed the screening; all subjects aged 30 years or older who had never undergone hepatitis screening in the database of the Chiayi County Health Bureau were invited for screening. All participants signed a consent form before blood sampling. Two biomarkers were detected: qHBsAg (Architect HBsAg; Finisklin Business Park, Sligo, Ireland) and HCV Ag (Architect HCV core antigen detection assay; Abbott Laboratories, Sligo, Ireland). The detection limit of qHBsAg was 0.05 IU/mL. HBV DNA levels were <2000 IU/mL in all subjects with <8 IU/mL. The best cutoff level of qHBsAg to predict HBV DNA <2000 IU/mL was 200 IU/mL, with sensitivity, specificity, accuracy, and positive and negative predictive values of 75.0%, 76.1%, 75.8%, 70.0%, and 77.9%, respectively [11]. Patients positive for HCV Ag were referred to clinics or hospitals for further evaluation and treatment.

Sex differences in the prevalence of qHBsAg and HCV Ag were compared using the chi-squared test. The descriptive statistics of HCV Ag-positive participants were compared using independent *t*-tests and chi-square tests for continuous and categorical variables, respectively. The Cochran-Armitage trend test was used to test the trend in age-specific prevalence. Geographical distribution and prevalence of qHBsAg and HCV Ag were determined using QGIS 3.6 software, with statistical analysis performed using SAS version 9.4 (SAS Institute, Cary, NC, USA), with statistical significance set as a *p* > 0.05.

## 3. Results

### 3.1. Distribution of qHBsAg in Chiayi County

**Figure 2:**
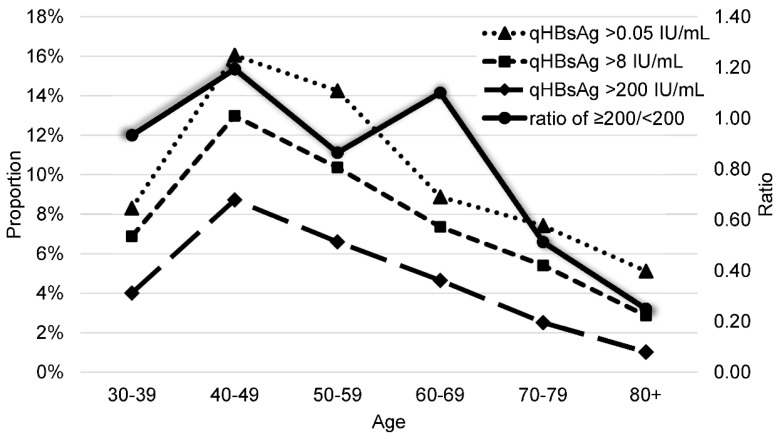
Age−specific prevalence of quantitative HBsAg by cutoffs of 0.05, 8, and 200 IU/mL. The ratio of ≥200/<200 was also shown.

A total of 4010 residents (male:female = 1630:2380) from 17 townships responded to the invitation for screening. The qHBsAg results are shown in Table 1, Figure 1 and Figure 2. The overall prevalence rate of qHBsAg was 9.9% (396/4010) without sex differences (males: 9.5% vs. females: 10.1%, *p* = 0.250). Among them, 76.8% (7.6%/9.9%) had qHBsAg ≥ 8 IU/mL and <50% (45.5% = 4.5%/9.9%) had qHBsAg ≥ 200 IU/mL. The distribution of age-specific prevalence was reverse U-shaped with the peak (16.0%, 68/424) at 40–49 years, and prevalence sharply decreased after this peak. The age-specific ratio of ≥200/<200 IU/mL was 0.85, ranging from 0.25–1.19, and the ratios markedly decreased after 70 years. Township-specific prevalence ranged from 6.3% to 21.1%. Alishan (21.1%, 24/114), Dongshih (17.4%, 30/172), and Budai (17.1%, 50/292) showed the highest prevalence. Taibao (12.6%, 25/198) and Puzi (10.2%, 29/285) were ranked 4th and 5th. Others were <10%.

### 3.2. Distribution of HCV Ag in Chiayi County

**Figure 3:**
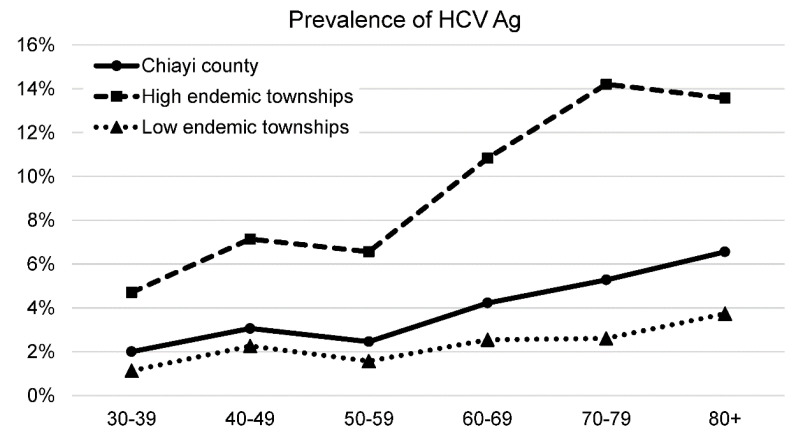
Age−specific prevalence of HCV Ag by endemicity of HCV.

The HCV Ag results are shown in Table 2, Figure 1 and Figure 3. The overall prevalence of HCV Ag was 4.1% (163/4010), without a sex difference (males: 4.2% vs. females: 3.9%, *p* = 0.665). Township-specific prevalence ranged from 0.3% to 14.6%. Lioujiao (14.6%, 40/274) and Yijhu (11.3%, 29/257) showed the highest prevalence rates. Sikou (7.2%, 11/152) and Dongshih (5.2%, 9/172) were ranked 3rd and 4th and others were <4%. The prevalence increased with age. The prevalence of the four previously mentioned high-prevalence townships were much higher than that those of the other 13 low-prevalence areas in each age group. The mean logarithmic concentration of HCV Ag was 3.32 ± 0.90.

### 3.3. Linkage to Care after HCV Ag Screening

A total of 163 patients were found to be HCV Ag-positive. Two patients died within three months after screening. Through telephone interviews, ten cases claimed that the report of the referral hospital was anti-HCV-negative and/or HCV RNA-negative. The false-positive rate was 6.3% (10/161). Among the residual 151 HCV Ag-positive subjects, 114 (75.5%, 114/151) underwent DAA treatment. Among patients aged ≥ 75 years, only 57.4% (31/54) underwent treatment. This was significantly lower than that in patients aged <75 years (85.6%, 83/97) (*p* < 0.001) (Table 3).

## 4. Discussion

Blood-borne diseases, including hepatitis B, C, and D, are endemic within special groups with active transmission routes, such as drug abusers. Geographic aggregation is an epidemiological phenomenon. Hepatitis B has a large-area geographic aggregation due to the vertical transmission of certain populations [18]. Hepatitis C showed geographic aggregation of different sizes due to iatrogenic transmission. The endemic areas may be as large as the entire country [19] or as small as a township or a part of a village [20]. Exposure to contaminated syringes and needles in early life has resulted in a small geographic aggregation among older people in some countries [20,21]. Egypt is a typical example [19]. This is also a common epidemiological pattern of HCV infection in Taiwan [3,20]. To maximize the effects of hepatitis C control, accurate epidemiological information is an important reference for a control program designed, especially for priority allocation of medical resources. This study identified two hyperendemic townships with HCV Ag prevalence >10% and three other endemic townships from this endemic county. Chiayi County should not be an HCV-endemic county. Based on these findings, the county government placed these two townships as the first priority for HCV control, although any epidemiologically based HCV control program should be promoted to other areas with a similar background.

In the era of effective DAAs in the treatment of hepatitis C, screening for anti-HCV is insufficient. Recently, some solutions have been published, such as the point-of-care HCV RNA test [22,23], anti-HCV reflex HCV RNA test [14], anti-HCV reflex HCV Ag test [15], and single HCV Ag test [14,24,25]. These tests can decrease unnecessary referrals and visits, which can reduce the burden on rural areas. HCV Ag testing alone is slightly more cost-effective than the anti-HCV reflex HCV RNA test [14]. A false-negative, that is, the test not being sensitive enough, was pointed out in the EASL guidelines [8]. In our community-based study, the negative predictive value was 99.3%. However, false positives are also a problem, and our recent study revealed that the positive predictive value was 98.4% [15]. Once HCV Ag is selected for screening, 1% false-negative and 2% false-positive results should be considered. It might be acceptable for large-scaled screening in rural areas; however, a certain proportion of false positives in early community-based [10] and hospital-based studies [26] was noted. The false-positive rate was high, and the cutoff was moved higher or in combination with the anti-HCV titer to resolve the problem at that time. In a recent study [14], the high false-positive rate of a local laboratory was still a problem. Using HCV RNA as a standard, all positive samples were retested in a central laboratory in a blinded manner. No false-positive subjects were noted in the well-controlled and experienced laboratory, as the high false-positive rate resulted from the operational technique. This large-scale study was conducted in local laboratories with a false-positive rate as high as 6.3%. With this experience, some technical advice was given to the less experienced and local laboratories to decrease false positives. The promotion of the anti-HCV reflex HCV Ag test for community-based studies was also initiated. Assuming that the prices of HCV Ag and anti-HCV were $20 and $7, respectively, with 1000 subjects with a 10% anti-HCV-positive rate, the cost of HCV Ag alone would be $20,000, while the anti-HCV reflex HCV Ag test would be ($7 × 1000) + ($20 × (1000 ×10%)) = $9000; therefore, the latter methods are more cost effective. As a general epidemiological rule, the false-positive rate should be lower in a population with a high prevalence. The probability of false positives should be decreased by limiting the HCV Ag to anti-HCV positivity. For these reasons, the anti-HCV reflex HCV Ag test was selected for the following community-based screening. Using the HCV Ag test, the quantitative concentration was obtained in IU/L. Based on a previous study, a significant, strong positive correlation between HCV Ag and HCV RNA was found (log (HCV RNA) = 0.957 × log (HCV Ag) + 2.735, R2 = 0.921, *p* < 0.001), with a correlation coefficient of 0.960 [27]. For example, in this study, the mean logarithmic HCV Ag was 3.32. The mean logarithmic concentration of HCV RNA can be estimated as 5.91, with the mean HCV RNA level ideally near 1,000,000 IU/mL. Since there is a high sustained virology response rate with current DAA treatment, the importance of HCV RNA concentration is ignored.

QHBsAg has been reported to correlate with serum HBV DNA for nearly 20 years [9]. Clinically, it has been used to monitor the treatment effect of interferons on chronic hepatitis B [28]. Recently, it has been used as a predictive marker for relapse after cessation of NUC therapy [29]. In a community-based study of the HBV cohort, it was used as a poor prognostic marker to predict the risk of hepatocellular carcinoma and as a good prognostic marker to predict HBsAg clearance [12]. Therefore, qHBsAg can provide more information on viral activity than qualitative HBsAg can. The most essential criterion for antiviral treatment of chronic HBV infection is an HBV DNA level > 2000 IU/mL. qHBsAg plays a role in predicting HBV DNA < 2000 IU/mL. In our community-based study, qHBsAg levels of 23% of carriers were <8 IU/mL, and all of their HBV DNA were <2000 IU/mL. The best cutoff level of qHBsAg to predict HBV DNA < 2000 IU/mL was 200 IU/mL, with sensitivity, specificity, accuracy, and positive and negative predictive values of 75.0%, 76.1%, 75.8%, 70.0%, and 77.9%, respectively [11]. In this study, we found that less than half of HBsAg-positive subjects had low HBV DNA concentrations, especially subjects aged 70 or older. Most cases with a lower viral load have a lower chance of developing liver cirrhosis or hepatoma [5,6,27], except in cases with advanced fibrosis [30]. With this additional information, we plan to arrange ultrasonography or FibroScan for HBsAg-positive subjects to allocate their risk. Despite infection or clearance, the peak HBsAg carrier rate was the highest in the youngest non-vaccinated cohort. On the basis of these findings, we plan to eliminate HBV after HCV elimination. The detection of qHBsAg ranges from 0.05 to 250 IU/mL. The cutoff values used for the prediction of HBV DNA < 2000 were 8 IU/mL and 200 IU/mL, respectively. No further dilution tests were required. The cost of the non-diluted qHBsAg test was almost the same as that of qualitative HBsAg.

Under the consideration of screening and linkage to care, staff of primary health care centers referred HCV Ag-positive cases to gastroenterologists or hepatologists. Outreach special clinics for DAA treatment were established in five townships without accessible treatment clinics. However, only 75.5% of patients were treated. This is slightly lower than the WHO requirement (80%). Old age was the most significant barrier; among patients aged ≥75 years, only 57.4% underwent treatment. Most patients were >60 years of age. Geriatric considerations are necessary when designing the accessibility of medical care.

This study had some limitations. In the study county, there are 357 villages in 18 townships. Our sample size was approximately 4000, which is sufficient to represent the prevalence of township levels and easily identifiable endemic townships. The study protocol might not be feasible at levels as small as those in the village setting. Although the positive and negative predictive values of HCV Ag were higher than 98%, a balance between feasibility and accuracy should be considered. Epidemiological baselines of both HBV and HCV were obtained, but only focused on HCV elimination by prescribing DAAs. Community-based HBV elimination programs for non-vaccinated cohorts should be designed in the near future.

An accurate epidemiological baseline is essential for medical resource allocation to eliminate HCV infection in areas with epidemiological patterns of community aggregation. A complete health database to prevent repeated screening and the design of feasible screening tools is essential to consider cost-effectiveness and consequent referral. To improve the accessibility of antiviral treatment, five outreach hepatological clinics were established in the township without a hepatologist. Much was learned from this study, and it is hoped that this program will be a useful reference for community-based HCV elimination programs.

Quantitative HBs Ag and HCV Ag as community-based screening strategies allow us to identify not only infectivity of HBV and HCV but also endemic regions. To improve the false-positive rate of HCV Ag screening, strategies like anti-HCV reflex HCV Ag are alternative methods for community-based screening.

## Figures and Tables

**Table 1:** Prevalence of quantitative HBsAg (qHBsAg) in different cutoffs grouping by sex, age, and township.

		qHBsAg (n/%)		
Groups	N	>0.05 IU/mL	>8 IU/mL	>200 IU/mL
Total	4010	396 (9.9%)	303 (7.6%)	182 (4.5%)
Sex				
Male	1630	155 (9.5%)	115 (7.1%)	64 (3.9%)
Female	2380	241 (10.1%)	188 (7.9%)	118 (5.0%)
Age (years)				
30–39	349	29 (8.3%)	24 (6.9%)	14 (4.0%)
40–49	424	68 (16.0%)	55 (13.0%)	37 (8.7%)
50–59	772	110 (14.2%)	80 (10.4%)	51 (6.6%)
60–69	1182	105 (8.9%)	87 (7.4%)	55 (4.7%)
70–79	795	59 (7.4%)	43 (5.4%)	20 (2.5%)
80+	488	25 (5.1%)	14 (2.9%)	5 (1.0%)
Township				
High prevalence (>17%)				
Alishan Township	114	24 (21.1%)	19 (16.7%)	10 (8.8%)
Dongshih Township	172	30 (17.4%)	24 (14.0%)	12 (7.0%)
Budai Township	292	50 (17.1%)	40 (13.7%)	24 (8.2%)
Medium prevalence (10~13%)				
Taibao City	198	25 (12.6%)	22 (11.1%)	16 (8.1%)
Pozi City	285	29 (10.2%)	22 (7.7%)	18 (6.3%)
Low prevalence (<10%)				
Yijhu Township	257	25 (9.7%)	20 (7.8%)	11 (4.3%)
Fanlu Township	226	21 (9.3%)	17 (7.5%)	8 (3.5%)
Jhuci Township	76	7 (9.2%)	3 (3.9%)	2 (2.6%)
Jhongpu Township	282	25 (8.9%)	18 (6.4%)	10 (3.5%)
Shueishang Township	316	27 (8.5%)	19 (6.0%)	11 (3.5%)
Singang Township	324	27 (8.3%)	20 (6.2%)	14 (4.3%)
Meishan Township	249	20 (8.0%)	18 (7.2%)	12 (4.8%)
Sikou Township	152	12 (7.9%)	9 (5.9%)	7 (4.6%)
Lucao Township	263	20 (7.6%)	14 (5.3%)	7 (2.7%)
Dalin Township	197	14 (7.1%)	10 (5.1%)	6 (3.0%)
Lioujiao Township	274	19 (6.9%)	17 (6.2%)	6 (2.2%)
Minsyong Township	333	21 (6.3%)	11 (3.3%)	8 (2.4%)

**Table 2:** Prevalence and mean logarithmic concentrations of HCV Ag and treatment rates of direct-acting antiviral agents (DAA) grouped by sex, age, and township.

Groups	N	HCV Ag (+)	log10 HCV Ag ^1^	DAA Therapy
Total	4010	163 (4.1%)	3.32 ± 0.90	114/163 (69.9%)
Sex				
Male	1630	69 (4.2%)	3.25 ± 0.92	52/69 (75.4%)
Female	2380	94 (3.9%)	3.36 ± 0.90	62/94 (66.0%)
Age (years)				
30–39	349	7 (2.0%)	3.14 ± 1.02	6/7 (85.7%)
40–49	424	13 (3.1%)	2.64 ± 1.33	6/13 (46.2%)
50–59	772	19 (2.5%)	3.28 ± 0.91	17/19 (89.5%)
60–69	1182	50 (4.2%)	3.52 ± 0.81	39/50 (78.0%)
70–79	795	42 (5.3%)	3.24 ± 0.92	29/42 (69.0%)
80+	488	32 (6.6%)	3.43 ± 0.67	17/32 (53.1%)
Township				
High prevalence (>11%)				
Lioujiao Township	274	40 (14.6%)	3.65 ± 0.78	26/40 (65.0%)
Yijhu Township	257	29 (11.3%)	3.40 ± 0.81	23/29 (79.3%)
Medium prevalence (5~8%)				
Sikou Township	152	11 (7.2%)	3.41 ± 0.93	7/11 (63.6%)
Dongshih Township	172	9 (5.2%)	3.26 ± 0.84	8/9 (88.9%)
Low prevalence (<4%)				
Jhuci Township	76	3 (3.9%)	3.81 ± 0.25	3/3 (100.0%)
Singang Township	324	12 (3.7%)	3.35 ± 0.81	7/12 (58.3%)
Jhongpu Township	282	9 (3.2%)	3.31 ± 0.99	9/9 (100.0%)
Lucao Township	263	8 (3.0%)	2.75 ± 1.06	5/8 (62.5%)
Taibao City	198	6 (3.0%)	2.64 ± 1.10	2/6 (33.3%)
Fanlu Township	226	6 (2.7%)	3.35 ± 1.17	4/6 (66.7%)
Alishan Township	114	3 (2.6%)	2.77 ± 1.24	3/3 (100.0%)
Dalin Township	197	5 (2.5%)	2.87 ± 0.36	3/5 (60.0%)
Minsyong Township	333	8 (2.4%)	2.45 ± 1.18	2/8 (25.0%)
Pozi City	285	6 (2.1%)	3.44 ± 1.01	6/6 (100.0%)
Shueishang Township	316	5 (1.6%)	3.22 ± 0.84	3/5 (60.0%)
Meishan Township	249	2 (0.8%)	3.52 ± 0.24	2/2 (100.0%)
Budai Township	292	1 (0.3%)	3.35	1/1 (100.0%)

^1^ Mean ± SD for HCV Ag-positive subjects.

**Table 3:** The age and sex of HCV Ag-positive patients detected in this screening by Direct-acting Antiviral Agents (DAA) treatment.

	Total (*n* = 151)	DAA Treatment (*n* = 114, 75.4%)	Without DAA Treatment (*n* = 37, 24.5%)	*p*-Value
Age	67.6 ± 13.4	66.1 ± 12.7	72.4 ± 14.5	0.012
<75	97	83 (85.6%)	14 (14.4%)	<0.001
≥75	54	31 (57.4%)	23 (42.6%)	
Sex				0.089
Male	63	52 (82.5%)	11 (17.5%)	
Female	88	62 (70.5%)	26 (29.5%)	

## Data Availability

All data generated and analyzed in this study are included in this article.
